# Analysis of Technological Innovation and Environmental Performance Improvement in Aviation Sector

**DOI:** 10.3390/ijerph8093777

**Published:** 2011-09-22

**Authors:** Joosung Lee, Jeonghoon Mo

**Affiliations:** 1 Graduate School of Innovation and Technology Management & Cho Chun Shik Graduate School for Green Transportation, Korea Advanced Institute of Science and Technology (KAIST), Daejeon 305-701, Korea; E-Mail: jooslee@kaist.ac.kr; 2 Department of Information and Industrial Engineering, Yonsei University, Seoul 120-749, Korea

**Keywords:** aviation energy use, environmental innovation, alternative fuels, biofuels

## Abstract

The past oil crises have caused dramatic improvements in fuel efficiency in all industrial sectors. The aviation sector—aircraft manufacturers and airlines—has also made significant efforts to improve the fuel efficiency through more advanced jet engines, high-lift wing designs, and lighter airframe materials. However, the innovations in energy-saving aircraft technologies do not coincide with the oil crisis periods. The largest improvement in aircraft fuel efficiency took place in the 1960s while the high oil prices in the 1970s and on did not induce manufacturers or airlines to achieve a faster rate of innovation. In this paper, we employ a historical analysis to examine the socio-economic reasons behind the relatively slow technological innovation in aircraft fuel efficiency over the last 40 years. Based on the industry and passenger behaviors studied and prospects for alternative fuel options, this paper offers insights for the aviation sector to shift toward more sustainable technological options in the medium term. Second-generation biofuels could be the feasible option with a meaningful reduction in aviation’s lifecycle environmental impact if they can achieve sufficient economies of scale.

## 1. Introduction

The past oil crises have caused dramatic improvements in fuel efficiency in all industrial sectors [1]. Automobiles, buildings, and other sectors invested in highly fuel-efficient systems and brought about energy-saving technological innovations [2]. The aviation sector—aircraft manufacturers and airlines—also made efforts to improve fuel efficiency through more advanced jet engines, high-lift wing designs, and lighter airframe materials [3].

However, it seems that the innovations in energy-saving aircraft technologies did not speed up, even during the oil crisis periods. The first oil shock was in 1973–1974 and the second one in 1978–1980 where periods in which only incremental improvements to aircraft technologies were introduced. The largest improvement in aircraft fuel efficiency was made in the 1960s while the high oil prices in the 1970s and on did not provide manufacturers or airlines with enough incentives to promote a faster rate of innovation [3]. Now with the backdrop of international concern on global warming, in addition to the rising oil prices, airlines are again keen to make energy-saving innovations in both technologies and operations.

Most attempts to better explain which factors drove innovations in the aviation sector have been technology-based approaches. Lee *et al.* [3] and several previous studies revealed that aircraft technologies improved quickly in the 1950s and 1960s (mostly jet engines) but the pace has slowed since the 1970s due to the limits associated with engine and aerodynamic efficiency improvement.

An economic explanation is that airlines passed the high fuel costs on to passengers so that the airfares rose during the 1970s and 1980s. The passengers were relatively insensitive to the ticket price because the conveniences of faster travel. As a result, the air travel volume rather increased during that period [4]. This is a very interesting trend since high fares are normally used to curb increasing traffic volume, but air passengers were willing to pay more for a faster mode of travel (and the resulting time savings).

Lee [5] also gave a society-driven explanation on why aircraft fuel efficiency improvement lagged that of other engineering systems or even aircraft noise reduction. It was revealed that the social awareness levels concerning the impact of jet engine emissions on climate change or local air quality was not sufficiently high; therefore, the aviation industry did not actively invest in truly innovative energy-saving technologies in aircraft systems.

Perhaps the combined reasons above generated the overall trend; therefore, we employ a historical analysis to examine the reasons behind the relatively slow technological innovation in aircraft fuel efficiency. The hypothesis is that the industry had low incentives to innovate in the past while green consumer awareness (green market) is low. As sustainability is now a new megatrend and industry is trying to make it a business strategy, and a future pathway to sustainable aviation will be discussed.

## 2. Aircraft Performance Improvement Trend

This section summarizes the key measures of aircraft performance and their recent trends from Lee *et al.* [6]. When judging the efficiency of an aircraft system, it is more relevant to consider work in terms of passengers or payload carried per unit distance. Energy intensity (E_I_) is an appropriate measure when comparing efficiency and environmental impact to other modes [7]. E_I_ consists of two components: energy use, E_U_, and load factor, α, as described by Equation (1) where MJ indicates mega joules of fuel energy and RPK stands for revenue passenger-kilometers and ASK for available seat-kilometers. Energy use is energy consumed by the aircraft per seat per unit distance traversed, and is determined by aircraft technology parameters including engine efficiency. E_U_ observed in actual aircraft operations reflects operational inefficiencies, such as ground delays and airborne holding. The fleet average E_U_ is of interest because it is the fleet fuel efficiency that determines the total energy use [6]. Load factor is a measure of how efficiently aircraft seats are filled and aircraft kilometers are utilized to generate revenue. Increasing the load factor leads to improved fuel consumption on a passenger-kilometer basis:

(1) $$E_{I}=\frac{MJ}{RPK}=\frac{MJ}{ASK}/\frac{RPK}{ASK}=\frac{E_{U}}{\alpha }$$

**Figure 1:**
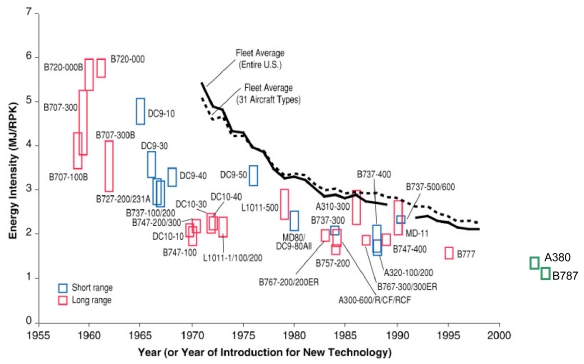
Historical trends in energy intensity of the US large commercial fleets. Individual aircraft E_I_ are based on 1991–1998 operational data with the exception of the B707 and B727, which are based on available operational data prior to 1991. Fleet averages were calculated using a revenue passenger-kilometer (RPK) weighting. Data was not available for the entire US fleet average during 1990 and 1991. Source: Lee *et al.* [3] modified by the authors.

Figure 1 shows historical trends in E_I_ for the U.S. large commercial fleet. Lee *et al.* [3] suggest that 57% of the reductions in energy intensity during the period 1959–1995 were due to improvements in engine efficiency, 22% resulted from increases in aerodynamic efficiency, 17% were due to more efficient use of aircraft capacity, and 4% resulted from other changes, such as increased aircraft size [3,8–12]. Year-to-year variations in E_I_ for each aircraft type due to different operating conditions, such as load factor, flight speed, altitude, and routing controlled by different operators, can be ±30%, as represented by the vertical extent of the data symbols.

We note the fast rate of innovation before 1960 and the slower pace since then. During the oil shocks in the 1970s, aircraft technological innovations were slowing down due to technological limits in conventional jet engines, wing design and airframe materials. The cost of innovation toward more radical forms of aircraft was enormous, so the aviation industry was reluctant to invest in such technologies [13]. It was more economical to pay for higher oil prices in the near term [3]. At the same time, it appears that either government or society did not strongly demand more energy-saving technologies.

Reductions in E_I_ do not always directly imply lower environmental impact. For example, the prevalence of contrails is enhanced by greater engine efficiency [14]. NO_X_ emissions also become increasingly difficult to limit as engine temperatures and pressures increase—a common method for improving engine efficiency [15]. These conflicting influences make it difficult to translate between the expected changes in overall system performance and the impact on air quality.

Engine, aerodynamic, and structural efficiencies play an important role in determining the energy intensity of an aircraft. Engine efficiency in large commercial aircraft, as measured by the cruise specific fuel consumption of newly-introduced engines, has improved by approximately 40% over the period 1959–2000, which equates to an average improvement of 1.5% annually [3]. Most of this improvement was realized prior to 1970, with the introduction of high bypass turbofan engines. However, as bypass ratios have increased, engine diameters have also become larger, leading to an increase in engine weight and aerodynamic drag [16]. Aerodynamic efficiency in large commercial aircraft has increased by approximately 15% historically, averaging 0.4% per year for the same period. Historical improvements in structural efficiency are less evident. One reason is that over the 35-year period between the introduction of the B707 and the B777, large commercial aircraft in service have been constructed almost exclusively of aluminum and are currently about 90% metallic by weight. Composites are used for a limited number of components. Another reason is that improvements in aircraft structural efficiency have been largely traded for other technological improvements like larger, heavier engines and increased passenger comfort [16,17].

If technological and operational improvements in aircraft systems continue to occur at a pace seen historically, a 30% to 50% reduction in E_U_ would be possible by 2025 [3]. This is equivalent to a 1.2% to 2.2% annual change in E_I_. Even though this is in line with industry expectations, such large improvements in technology may not be reached within the next 15 years. For example the 747–400 entered service in 1989, while the new A380 arrived almost exactly 20 years later. The E_I_ of the new Airbus A380 is 12% lower with respect to the B747-400 [18], which makes 0.7% improvement per year. The annual reduction is not constant but diminishing because of increasing cost and time to develop better technology. Note that this pace of change is not sufficient to counter the projected annual 4%–6% growth in demand for air transport. Unless measures are taken to significantly alter the dominant historical rates of change in technology and operations, the impacts of aviation emissions on local air quality and climate will continue to grow.

## 3. Drivers of Aircraft Technology Innovation

Historically, three major drivers have existed for aircraft technological innovation. One major driver was fuel cost. Previous studies [6,16] examined the improvement in aircraft fuel efficiency. Since fuel costs account for 20% to as high as 50% of the direct operating cost of aircraft, both manufacturers and airlines are highly interested in fuel-saving technologies that reduce their operating cost.

The second type of driver is the current movement on global climate change and sustainability. Over the past 10 years, the aviation sector has received attention regarding the jet emissions’ potential impact on global warming and local atmosphere [19]. As sustainability has become a major issue for the aviation sector, both aircraft manufacturers and airlines are focused on technological and operational means to mitigate aviation’s climate impact.

The third type of innovation driver for aircraft performance is social demand. Aircraft noise was known to cause hearing impairment in the 1960s and public demand for quieter aircraft accumulated over time. Governments responded by phasing out noisy aircraft and eventually today’s aircraft are several times quieter than earlier jet aircraft [20]. On the contrary, aircraft fuel economy, which is a surrogate measure of jet engine emissions (mostly CO_2_), improved slowly during the same period. One factor for this phenomenon is the low level of social awareness on aviation and climate issues [21].

The next subsections examine these drivers in detail. Then the question of why aircraft technological innovation is slow is analyzed in the subsequent section.

### 3.1. Economic Driver: Fuel Cost–Passenger Volume Relationship

Historically, fuel cost has been the main driver for improvements in aircraft fuel efficiency. Note that fuel efficiency in itself is not a goal of aircraft design but a means to achieve other targets like speed, payload-range, landing/takeoff performance. Fuel efficiency gain was strongest during the 1960s when oil prices make up a large portion of airlines’ direct operating cost [6,16]. When oil prices soar, airlines actively adopt advanced aircraft with greatly improved fuel economy. Note that there are other important reasons for the aircraft designers to develop fuel efficient aircraft regardless of fuel costs. Fuel efficiency has a strong impact on major design objectives like payload-range performance and landing takeoff performance of an aircraft. Every 100 kg of fuel saved may add an extra passenger on a given weight-limited range. Also ‘hot and high’ airfields limit takeoff weight for specific flight-legs and every kg of fuel saved helps to reduce such constraints. This is a fundamental difference with cars, ships and trains, where weight and volume are constraining the design to a significantly less extend. These cause aircraft designers to have a focus on fuel efficiency, even when fuel prices are expected to be low in the future.

**Figure 2:**
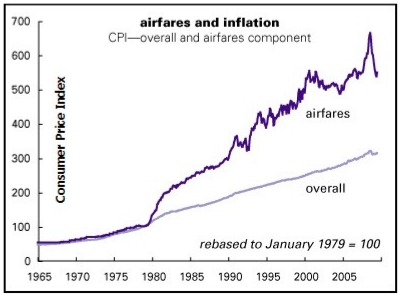
Historical airfares and inflation. Source: Henwood [22].

To understand how increased oil prices impacted airfares and subsequent air travel, Figure 2 shows a historical trend in airfares since 1965. The first oil shock did not cause airfares to go up since the airline industry was still regulated [22]. The second oil shock’s impact is clearly seen by the jump in airfares. Deregulation took place in 1978, after which airlines could charge fares of their own and decide schedules and routes by maximizing profits. Thus, the second oil shock caused airfares to jump up, the rate of which was greater than that of inflation [23]. The jump in fares after deregulation was not only because of the high oil prices, evidenced by the fact that fares stayed high afterwards. It had a lot to do with deregulation itself as airlines could now set their own fares, and could charge what the market would pay, which was apparently higher than the regulated fares.

**Figure 3:**
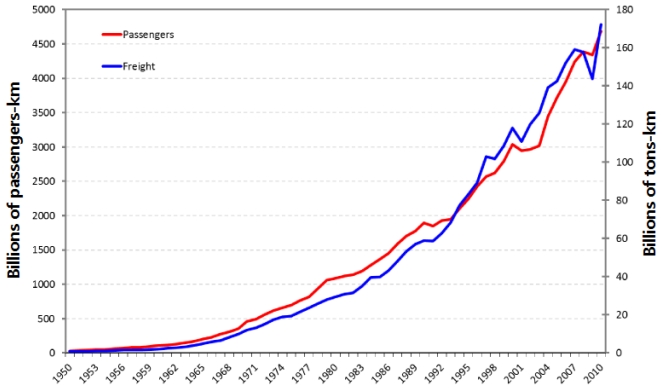
Historical trend in air traffic volume. Source: International Air Transport Association (IATA) [24].

An interesting trend is that air travel volume rather increased while airfares rose, regardless of high oil prices. Figure 3 shows that air travel demand increased continuously throughout the 1970s and 1980s. Passenger and freight volume showed a visible dip only in the early 1990s due to economic depression and intense competition [24] and after September 11, 2001. Surprisingly, air travel did not decrease during the two oil shocks. This shows that the convenience of air travel continued to attract air travelers. To be more specific, the first oil shock did not cause airfares to increase so the passenger volume did not shrink. The second oil shock, however, did pull up airfares, but people continued to fly because air travel enabled significant convenience in long distance and leisure travel [4].

**Figure 4:**
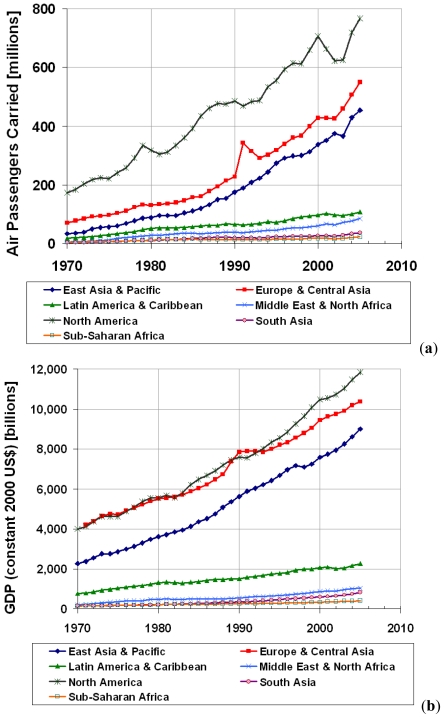
Trends in air travel volume and income growth. (**a**) Air passengers carried by airlines registered in those regions; (**b**) GDP (constant 2000 US$): country aggregates by regions. Source: Ishutkina and Hansman [25].

To further explain why airfares and air travel volume increase together, we must examine trends in people’s disposable income. Figure 4 shows air travel increases quite linearly with respect to gross domestic product (GDP). The evolution of this passenger transport is driven by two factors. One is the travel money budget, which indicates that humans dedicate a fixed share of their income to travel. The other factor is the travel time budget, which describes that humans spend an average of 1.1 hours on travel per day in a wide variety of economic, social, and geographic settings. Thus, human mobility rises as income level rises while the constant travel time budget pushes people towards faster transport modes as their demand for mobility increases [4]. As a result, continuing growth in world population and GDP are expected to lead to a high growth in air travel demand in the future, due to the convenience and time saving associated with air transport. If the strong growth in air travel continues, world air traffic volume may increase up to five- to twenty-fold by 2050 compared to the 1990 level and account for roughly two-thirds of global passenger-miles traveled [4,10].

When passengers were willing to pay more even under higher fuel costs and airfares, manufacturers or operators had relatively less incentive to invest in radical technological innovations because they were guaranteed sustainable revenues. Furthermore, the cost of switching to a radically improved technology (e.g., non-fossil fuels) has been too high and takes too long for development. Innovations in aircraft technology are hindered by the relatively long lifespan and large capital and operating cost of individual aircraft, and the inherent lag in the adoption of new technologies throughout the aviation fleet as a result. It has typically taken 10–15 years for the US fleet to achieve the same fuel efficiency as that of newly introduced aircraft. This process of technology uptake depends on various cost factors and market signals. In assessing future aviation fuel consumption and emissions, it is important to consider this time delay between technology introduction and its full absorption by the world fleet. Furthermore, the development programs for new aircraft typically begin 7–10 years before the inaugural aircraft is certified, and basic research required to support the new technology typically precedes the beginning of the development programs by several years. Thus, the time required for ideas to make the transition from basic research to fleet impact can be as much as 25 years. Perhaps most importantly, the cost of change is uncertain. Airlines are willing to pay higher acquisition costs if they can save in direct operating costs, mainly through lower fuel and maintenance costs during the lifetime of aircraft. However, it is unclear whether future technologies can be delivered at an acceptable price. If the price is too high, airlines may choose not to pay more for energy saving technologies, in which case further improvements in energy use for the aviation sector may be limited. As a result, the air transport industry invests only in modest innovation in fuel-saving technology and operations [3].

In sum, aircraft innovations slowed down since the 1970s due to the slower pace of technological advancement in engine design. Aerodynamic design and airframe materials improved, but at a slower pace, too. In other words, engine, aerodynamic and structure technologies are approaching the limits of physics and thus become more and more difficult to achieve (at higher cost and time investments). Thus this trend necessitates introduction of more radical technological and operational measures. The long leadtime in product development and fleet turnover as well as the high cost associated with radical technological breakthroughs was another barrier. Note that airlines order more fuel-efficient aircraft when fuel costs are high, but the delivery only comes a few (in some cases quite a few) years later, so they are not able to respond immediately by buying new aircraft. They can, however, retire older aircraft, as was seen recently with the retirement of many of the MD-80s in the US when the oil price peaked. Accompanying this trend was the passenger’s willingness to pay higher fares as a result of increased income and the convenience of air travel.

### 3.2. Sustainability Driver: Environmental Considerations Changing the Scene

Growth in the total volume of air transportation has important environmental ramifications associated with climate change and stratospheric ozone reduction on a global scale. On local to regional scales, issues such as noise, decreased air quality (related primarily to ozone production and particulate levels), roadway congestion (related to airport services), and local water quality are recognized as important consequences of air transportation. With the consumption of a dwindling fossil fuel supply, there is more attention than ever before on the emissions that aircraft produce [19].

Aviation fuel burn is responsible for approximately two to three percent of global carbon dioxide (CO_2_) emissions, and aviation it is considered to be a fastest growing, potentially significant source of greenhouse gas emissions [26,27]. Globally, aviation accounts for approximately four to nine percent of the climate change impact of anthropogenic activities. As demand for passenger and cargo air transportation continues to rise, the reduction of aviation’s environmental footprint becomes even more critical [28].

Although modern aircraft are much more eco-friendly than their predecessors, the number of airplanes in operation is growing and reducing jet engine emissions is more important with the sheer volume of air travel predicted for the future. Commercial aviation is increasingly being targeted by legislators for mandatory carbon-trading schemes and limits on aircraft emissions [29]. With global economic downturn and an increased focus on environmental concerns, airlines are scrutinizing every step of their operations for new ways to gain efficiencies and cut their fuel bills. The financial pain of economic downturn and soaring fuel prices are particularly acute for airlines because fuel is their single biggest expense. Eight years ago, 15 percent of airfare went to pay for jet fuel; now, it is 40 percent, according to the Air Transport Association. Many airlines are already implementing measures to cut emissions and make their operations green. Aiming for further improvement, airlines have pledged to increase fuel efficiency by another 25 percent by the year 2020 [30].

The rapid increase in air travel demand, fuel consumption, and associated emissions has given rise to a global dialog to address the potential impact of aviation on climate change. Since 1977, a United Nations’ specialized agency, International Civil Aviation Organization (ICAO) has promulgated international emissions and noise standards for aircraft and aircraft emissions through its Committee on Aviation Environmental Protection (CAEP). ICAO has also developed broader policy guidance on fuel taxation and charging principles. In protecting local air quality in the vicinity of airports, the U.S. first introduced legislation to set domestic regulation standards. ICAO subsequently developed International Standard and Recommended Practices for the control of fuel venting and of emissions of carbon monoxide, hydrocarbons, nitrogen oxides and smoke from aircraft engines over a prescribed landing/takeoff (LTO) cycle below 3,000 feet. While there is no regulation or standard for aircraft emissions during cruise, these LTO standards also contribute to limiting aircraft emissions during cruise. The Kyoto Protocol’s Article 2 contains the provision that industrialized countries pursue policies and measures for limitation or reduction of greenhouse gases from aviation bunker fuels. In relation to other aircraft engine emissions, IPCC has underlined the continuing uncertainties associated with the impacts of nitrogen oxides, water vapor, and sulfur while asking for further research [10].

The ICAO’s CAEP is primarily responsible for monitoring the aviation industry’s emissions and noise reduction efforts and seeking further options to mitigate the impacts of aviation on community noise, local air quality and the global atmosphere. Over the years, CAEP has set aircraft engine certification standards and phase-outs of noisy aircraft [31]. Many options for emissions mitigation have been proposed, including higher fuel taxes, emission charges, emission caps or limits, emissions trading, increased stringency of the certification standards, retrofit mandates, voluntary actions, demand management, and the possibility of no action. Although negotiations will settle the form of such policies, they all fundamentally entail considerations of the efficacy of both technological and operational strategies for system efficiency improvement and emissions reduction [3].

### 3.3. Social Driver: Pubic Demand

The external factors that can influence the transition arena in aviation industry present future scenarios for the commercial aviation paradigm. These are the necessity to change the aviation industry towards a more sustainable aviation paradigm and technological innovation. The necessity is a result of a global perception of the environment and is influenced by society as a whole. Technological innovation is the more immediate and tangible factor since it directly influences the industry’s technological development. It can be seen that technological innovation is not only driven from innovators but also influenced by society. The present situation represents lowest scores on both factors [13].

In order to achieve continued improvements in aircraft fuel efficiency, as well as reductions in jet engine emissions that adversely impact global climate and local air quality, there should be a stronger social pressure on the aviation sector. That is, social pressure sends a signal to governments that something is worrisome for human health and the environment. Governments then take action (either via a command-control or incentive-based mechanism) after confirming scientifically the cause of the problem as well as the solution. Currently, the social demand for low-emission aircraft is not strong enough because the general public is not well aware of the effects of aviation emissions on the global climate [32,33]. Note that in some countries (e.g., UK) the press has targeted aviation to a far greater extent than other industries, and the aviation industry in fact feels that there is far more public awareness than is warranted (aviation being only 3% of anthropogenic CO_2_ emissions). However, in other regions like Asia where air traffic is significantly increasing, public awareness is still very low. At the same time, the effects of aircraft engine emissions on the global atmosphere are not well understood scientifically.

The previous section discussed that the primary motivation to improve aircraft fuel economy has been to lower fuel cost, and that aircraft manufactures and airlines are increasingly more conscious of global climate change due to jet engine emissions. However, scientific knowledge and public awareness about the impacts of aviation emissions on the global atmosphere are still low. This is the key difference from the case of aircraft noise reduction, where the scientific evidence and strong public demand have induced a large decline in aircraft noise levels. Historically, strong public demand supported by scientific evidence of health damage caused by aircraft noise and subsequent government regulation to limit the operation of noisy aircraft have led to large reductions in noise around airports. To examine this difference quantitatively, Lee [5] examined the social factors that drive environmental innovation in aircraft systems.

To expedite environmentally conscious innovations for low-emission aircraft, increased amounts of knowledge and information should flow between aviation firms and other societal constituents, such as citizens, governments, and civilian organizations. Knowledge accumulation is important in two aspects. One is that it provides a credible basis for the existence of the environmental problem. The other important aspect of knowledge is to provide the scientific capability to solve environmental problems. It is necessary that basic scientific knowledge be accumulated in order for firms to perform further research and develop new products [34]. Note that large uncertainty still exists in aviation climate science so this discourages industry from taking risks/costs associated with more environmentally friendly technological innovations.

The major role of information diffusion is to better inform the general public about the importance of environmental conservation through media including television, radio, newspapers, magazines, bulletins, and films. Events such as environmental week and school education programs are also good methods of information diffusion [35]. To raise the public awareness level (*i.e.*, information diffusion), much more active dissemination of and education about the environmental effects of aircraft engine emissions are needed [33]. Some European environmentalists are pushing for programs that increase awareness of air travel passengers, along with their sense of responsibility for global environmental protection. The programs enable passengers to pay a fee to mitigate their share of the damage from the carbon dioxide emitted during each flight. Environmental companies then use the money to plant trees, which remove carbon dioxide from the air. British Airways has an ‘emissions calculator’ on its website so that passengers can determine how much carbon dioxide is emitted due to their flying [35]. Note that some experts pose objections against this idea. First there is simply no room for compensation through forests or other sectors as soon as aviation “takes up the full sustainable carbon footprint by 2040–2050” [27]. Second environmental consciousness will not be enhanced in a way that it will help consumers to adopt a less hypermobile lifestyle because compensations will appear to them to be a cheap way out of the personal dilemma [36,37].

The emissions calculator can “nudge” consumers to behave more environmentally consciously [38]. The problem is that air travelers do not seem to have simple means to behave environmentally consciously. The only way is that they travel less, but this will not be the case for most passengers (*i.e.*, they don’t give up vacation travel with cheap airline ticket deals [39]). Government and industry must work together to design practical means for air travelers to behave in an environmentally conscious manner.

All of these activities pertaining to knowledge accumulation and information diffusion will help construct an environmentally conscious market for the aviation sector and eventually give aviation firms a corporate social responsibility to adopt environmental performance improvement as part of their business strategy.

## 4. A Pathway to Sustainable Aviation

In this section, we examine advanced aircraft technologies and alternative fuels and analyze their technical and economic feasibility to achieve air transportation industry’s sustainable growth.

### 4.1. Alternative Technology Choices for Reduced Energy Use and Environmental Impact

The highly energy efficient aircraft, Boeing 787, is expected to use 20% less fuel than its contemporary counterparts. The key technologies include light-weight structures, highly efficient engines, and aerodynamic improvements to the body and wings. As much as 50 percent of the primary structure (including the fuselage and wing) on the B787 will be made of composite materials. The advanced engines for the new airplane are expected to contribute as much as 8 percent of its increased efficiency. According to the Boeing Company [40], it will be possible to eliminate 1,500 aluminum sheets and 40,000 to 50,000 fasteners by manufacturing a one-piece fuselage section, and to attain greatly improved aerodynamic and structural efficiency. Operational measures such as single-engine taxi are exercised actively in order to reduce fuel consumption and environmental impacts from jet engine emissions. Now both aircraft manufacturers and airlines are investing in more aggressive innovations. Future aviation CO_2_ emission share depends upon energy use and fuel mix of aviation relative to those of other sectors.

In the next decade, alternative fuels will be available to reduce aviation’s impact on climate although supplies are limited. Biodiesel and biokerosene have been suggested to be blended with conventional jet fuel; however, MIT research [41] indicated that neither fuel is appropriate for use in aviation. Even in light (*i.e.*, low-concentration) blends, these fuels may compromise safety during storage or during flight, leaving deposits in fuel systems. Tests of biodiesel light blends indicated freezing at typical operating temperatures. Ethanol is not suitable for aviation either. It has a low flash point and has high volatility, making it dangerous to handle and posing a risk to crew and passengers during flight. Moreover, its energy content per unit mass and per unit volume is approximately 40 percent less than that of jet fuel; therefore, flight range would be reduced and the amount of energy used to fly a given distance would increase relative to Jet A. These issues are not present when ethanol is used in ground-transportation applications [42].

The alternative aviation fuels that have the greatest production potential and environmental benefits over the next decade are as follows: (1) Fischer-Tropsch (FT) synthetic fuel produced from coal, a combination of coal and biomass, or natural gas; and (2) Hydroprocessed Renewable Jet (HRJ) fuel produced by hydroprocessing renewable oils. All three are or can easily and inexpensively be made fully compatible with current aircraft and fuel-delivery systems [41].

The prospects for FT jet fuels depend crucially on the construction of pilot plants in the next few years while production of commercial quantities of HRJ depends on the availability of appropriate feedstocks at competitive prices. For HRJ to be effective in reducing greenhouse gas (GHG) emissions, it must be produced from oils that do not incur land-use changes, either directly or indirectly, that cause a large release of other GHGs. This constraint places a severe limit on the amount of climate-friendly HRJ that can be produced within the next decade. For FT jet fuels to be effective agents for GHG reduction, they must be produced from biomass or a combination of coal and biomass. In the former case, the fuels will be expensive and demand extensive cultivation of biomass for inputs. In the latter case, capture and sequestration of plant-site carbon emissions would be required, but overall costs would be much less, as would biomass consumption. As with HRJ, the provision of biomass must not incur land-use changes, either directly or indirectly, that cause a large release of GHGs [41].

The aviation industry is therefore interested in developing fuels that can be mass produced at a low cost and high yield with minimal environmental impact. The extensive use of first-generation feedstocks will incur land-use changes that will cause a large increase in GHG emissions. Next-generation biomass feedstocks are needed that do not compete with food production and that consume little fresh water. “Second-generation” biofuels should be made from crops that are fast growing plants that do not take up productive arable land; do not require excessive farming techniques or threaten biodiversity; provide socio-economic value to local communities and importantly result in a lower carbon footprint. They include bio-derived oil, sourced from feedstocks such as jatropha, camelina, algae and halophytes, which can be mass grown in locations almost worldwide, including in deserts and salt water [28].

These biofuels are still anticipated to provide an estimated 80% reduction in overall CO_2_ lifecycle emissions compared to fossil fuels. For example, analysis of camelina feedstock use for aviation has shown even better results, with an 84% reduction in lifecycle emissions. Furthermore, biofuels contain fewer impurities (such as sulphur), which enables an even greater reduction in sulphur dioxide and soot emissions than present technology has achieved [28].

The target is to certify aviation biofuels by 2013, although there is now a possibility that a 50/50 blend of biofuels mixed with Jet A-1 fuel could be certified in the next year. Due to recent advances in research and technology, aviation biofuel might be available for commercial use within five years, once the feedstock production process has been set in motion [28].

Beyond the evolution of the current aircraft platform, hydrogen has been proposed as an alternative fuel for future low-emission aircraft [43]. Hydrogen-fueled engines generate no CO_2_ emissions at the point of use, may reduce NO_X_ emissions, and greatly diminish emissions of particulate matter. However, hydrogen-fueled engines would replace CO_2_ emissions from aircraft with a three-fold increase in emissions of water vapor [44,45]. In addition, there are several issues that must be resolved before a new fuel base is substituted for the existing kerosene infrastructure. While liquid hydrogen (LH_2_) can be used as a direct fuel in a combustion engine or it can be used for fuel cells to create electricity, it will require significant amounts of energy for its creation and storage [13]. The industry needs to overcome significant technical challenges in designing a hydrogen-powered aircraft for commercial aviation and in producing enough hydrogen in a sustainable way to supply the industry’s needs [28]. The usefulness of such alternative fuels requires a balanced consideration of many factors, such as safety, energy density, availability, cost, and indirect impacts through production. Some experts believe nuclear-powered or solar passenger aircraft are the option to propel future air transport systems. However, this will require a major research program to help the aviation industry convert from fossil fuels to such radically different energy [46,47].

The radical technological changes would not all be welcomed by existing regime players. Airport operators would not be satisfied with substituting the current fuel supply with alternative sources since there needs to be change in the current infrastructure. Kivits *et al.* [13] notes, “It is important for the aviation industry, and airports in particular, to understand what impact technological transition will have on existing infrastructural systems. The aviation industry has always relied on the presence of oil as its main fuel source, and a clear path dependency is in effect. The substitution of aviation energy from petroleum to another source will have a severe impact on the current supporting technology. A new energy paradigm may require a new distribution network, a new way of generating the fuel, a new type of engine, and perhaps even a new design of aircraft. It is quite possible that an unmentioned (or hitherto un-invented) technology could become the new world standard. No matter which technology gains the upper hand, the aviation industry, and its stakeholders by extension, must be ready to adapt to this technology and provide the requisite infrastructure. In general, airport operators have invested large sums of money in existing infrastructure, which is closely aligned to the requirements of current airplanes optimized for using Jet-A fuel. Since airports are largely dependent on airlines making use of their facilities, there is a formal relationship between the airports and the airlines, usually in the form of a contract whereby airlines rent slots and space. When airlines decide to use new types of airplane, airports can decide to modify airport infrastructure as required, if they feel that doing so it will ultimately benefit them. This occurred with the introduction of the B747 and the A380, which resulted in airstrips having to be elongated and terminals having to be adapted. However, since these infrastructural changes require substantial investments, airports will obviously not make these decisions lightly.”

Airlines will also favor incremental technological change due to large costs involved in selling old airplanes and getting new ones. Airplane manufacturers will not favor radical technological change as well since they have to design and build a completely new aircraft to optimize the new technology. For these reasons, it seems that substitution of fossil fuel by biofuels will be more favored from the industry given the cost and time for development [13].

### 4.2. Achieving Economic Viability of Alternative Fuels for Sustainable Aviation

The transition from jet fuel to biofuel has been analyzed by a game-theoretic approach used by Maciel *et al.* [48]. The game could be cooperative, when participants negotiate contracts, or non-cooperative, when agreements are not possible [49]. The analysis could be accomplished by observing the interaction between the conventional jet fuel and biofuel, thus simulating gain and loss scenarios for each player in the aviation industry. Thus far, the actions and strategies undertaken by each industry are qualitatively analyzed, especially in regard to the conventional jet aircraft industry. If there is cooperation for a biofuel-kerosene blend, then both will win with positive marginal benefits to the entire society. This scenario is where the industry will reach a consensus, and this biofuel-kerosene blend will be in the industry’s short-term best interests, allaying stakeholder concerns and satisfying shareholder requirements including those of government and the community. This results in the implementation of a technology of medium sustainability, requiring a minimal amount of infrastructural changes. This is the best option in terms of monetary resources over the short to medium term, but could prove undesirable in the long term since a more substantial technology breakthrough will be needed in the future. This work is on-going research, and its final results will be published in a subsequent article.

The conditions to achieve the economic viability of second-generation biofuels are discussed below. First, it is important that biofuel technologies mature, and the production achieve economies of scale in order to be cost competitive against conventional jet fuel. Some estimates indicate that biofuels will be commercially viable when they reach 1% of the total jet fuel supply. (*i.e.*, 10% of the world’s aircraft fleet is running on a mix of 10% biofuel and 90% Jet A-1). It is estimated that 85% of biofuel production costs relates to the cost of the feedstock. As technology to harvest and process these feedstocks progresses and as they become available in commercial quantities, the price will drop [28].

Owing to their renewable nature, these feedstocks can continue to be produced, over and over again. The price of oil can vary substantially, falling from a high of USD$147 per barrel in June 2008 to $40 in December 2008. This makes it difficult to project when biofuels would be competitive, but there are strong indications that biofuels would become cost-comparable with traditional jet fuel, Jet A-1, by around 2020. If industry considers additional costs of using fossil fuels in addition to the price of the fuel itself, the economic viability of biofuels can come sooner. Note that legislation passed by the European Union in 2008 to include aviation in the EU’s emissions trading scheme (ETS) will add a carbon cost to aviation, requiring airlines to pay for their carbon emissions from 2012 [28].

It is possible that emissions trading schemes will also be developed in other parts of the World. This makes alternative fuel technologies, which reduce emissions compared to traditional jet fuel, especially attractive. Under the European ETS legislation, biofuel use is zero-rated for emissions. Other policies could reduce tax levels on low-carbon fuels such as biofuel. The United States and other governments are on course to make significant investments in sustainable biofuel development. The industry has called on governments to assist potential biofuel suppliers to develop the necessary feedstock and refining systems–at least until the fledgling industry has achieved the necessary critical mass [28]. The positive incentives required include:

- Assistance in identifying the most suitable areas in which to grow these crops;
- Support in starting the farming and production of algae (e.g., building of facilities, hiring labor resources, buying seeds and setting up any irrigation components);
- Incentives for companies to develop the processing and refining capacity needed to turn raw feedstock into biofuel crude oil and then into biojet fuel;
- Positive fiscal and legal frameworks to facilitate the economic viability of these new types of fuels so that airlines can use them as quickly as possible.

Regarding the last type of incentive, a volume of biofuel, equivalent to the contracted amount, would be guaranteed to enter the aviation fuel supply chain somewhere in the world but would not necessarily be used by the contracting carrier. The carrier would, however, receive the benefit of any carbon savings associated with the cultivation of the fuels (e.g., a 50% saving compared with conventional jet fuel) and could include this in their carbon reporting at the end of a trading period [50].

## 5. Conclusions

The aviation sector’s fuel efficiency improvements have slowed down since the 1970s due to the slower pace of technological advancement in engine and aerodynamic designs and airframe materials. The long leadtime in product development and fleet turnover, as well as the high costs associated with radical technological breakthroughs were also major barriers. Accompanying this trend was the passengers’ willingness to pay higher fares as a result of increased income and the convenience of air travel. While aircraft manufactures and airlines are now increasingly more conscious of global climate change due to jet engine emissions, scientific knowledge and public awareness about the impacts of aviation emissions on the global atmosphere are still low. This is the key difference from the case of aircraft noise reduction, where strong public demand supported by scientific evidence of health damage caused by aircraft noise and subsequent government regulation to limit the operation of noisy aircraft have led to large reductions in noise around airports. Therefore, to expedite environmentally conscious innovations for sustainable air transportation sector, increased amounts of knowledge and information should flow between aviation industries and societal constituents, such as citizens, governments, and civilian organizations.

Based on the analysis of various alternatives for sustainable aviation, a meaningful reduction in environmental impact can be achieved through biofuel in the near future. Technological breakthrough will take a long time for development and diffusion, mainly due to cost of development and passenger’s actual willingness to pay more for the environment. Operational change is most near-term, cost-effective option, but it may not achieve a significant option given the fast increase in air travel demand.

An optimal pathway to sustainable aviation is possible by building high consensus and high perceived need among stakeholders (*i.e.*, industry, government and passengers). With a high perceived need, it is easier to commit more resources to research and development of sustainable solutions. The joint effort among the major stakeholders could lead to a fast and robust transition in aviation technology. It will be likely that such work will lead to significant infrastructural changes, the costs of which would have to be borne by all players within the transition arena.
